# Performance of an Immunochromatographic Test (ICT) in Comparison to Some Commonly Used Serological Tests for the Diagnosis of Brucellosis in Dromedary Camels (*Camelus dromedarius*)

**DOI:** 10.3390/microorganisms7120591

**Published:** 2019-11-20

**Authors:** Wissam S. Serhan, Rashid A. Khan, Esmat F. Gasim, Mariam S. Alketbi, Fabrizio De Massis, Paolo Calistri, Armando Giovannini, Mohamed A. Al Hosani, Saleha A. Al Jaberi, Asma M. Al Mansoori, Asma S. Al Ketbi, Abdelmalik I. Khalafalla, Salama S. Almuhairi

**Affiliations:** 1 Veterinary Laboratories Division, Abu Dhabi Agriculture and Food Safety Authority (ADAFSA), Abu Dhabi 52150, UAE; wissam.sobhi@adafsa.gov.ae (W.S.S.); Rashid.Khan@adafsa.gov.ae (R.A.K.); esmat.faisal@adafsa.gov.ae (E.F.G.); mariam.s.alketbi@adafsa.gov.ae (M.S.A.); mohamed.a.alhosani@adafsa.gov.ae (M.A.A.H.); saleha.aljaberi@adafsa.gov.ae (S.A.A.J.); asma.almansoori@adafsa.gov.ae (A.M.A.M.); Asma.AlKetbi@adafsa.gov.ae (A.S.A.K.);; 2 OIE Reference Laboratory for Brucellosis, Istituto Zooprofilattico Sperimentale dell’Abruzzo e del Molise “G. Caporale”, Campo Boario, 64100 Teramo, Italy; f.demassis@izs.it (F.D.M.); p.calistri@izs.it (P.C.); a.giovannini@izs.it (A.G.)

**Keywords:** brucellosis diagnosis, dromedary camels, serological tests, performance

## Abstract

Serological tests may represent an essential tool for the diagnosis of camel brucellosis; however, concerns arise in the scientific community regarding the direct transposition from cattle and small ruminants without adequate validation. The present study was made to compare four serological tests for the diagnosis of brucellosis in dromedary camels (*Camelus dromedarius*). In terms of sensitivity, our results show that the Immunochromatographic Test (ICT) shows the higher value of sensitivity, 98.67% (95% Confidence Level (C.L): 94.36–99.99%), followed by the Fluorescence Polarization Assay (FPA) with 95.05% (95% C.L: 88.23–99.51%), then the Competitive Enzyme-Linked Immunosorbent Assay (c-ELISA) with 94.94% (95% C.L: 88.25–99.45%) and, finally, the Rose Bengal Test (RBT) with 68.95% (95% C.L: 56.55–80.69%), which is the only test showing a significantly lower sensitivity compared to the others. On the other hand, our study revealed no significant difference in terms of specificity between all the tests under study, with a range from 99.06% (95% C.L: 98.34–99.64%) for the ICT to 99.92% (95% C.L: 99.64–100%) for the RBT. The ICT was found to be comparable in terms of sensitivity and specificity with the most commonly used tests for camel brucellosis. The results of the present study are of paramount importance for designing surveillance and control measures for brucellosis in camel populations.

## 1. Introduction

Brucellosis is a contagious zoonosis disease caused by a facultative intracellular Gram-negative coccobacillus known as *Brucella*. In livestock, brucellosis is considered as a high threat due to its negative impact on animal health, public health, and international trade [1,2]. The disease can be transmitted between animals by direct contact, products of conception, body fluids, contaminated environment, contaminated water, feed and raw milk [3]. Furthermore, brucellosis can also be transmitted to humans through the inhalation of aerosolized bacteria, contact with contaminated tissues and ingestion of the contaminated animal products [4].

Brucellosis in camels is caused mainly by two species of *Brucella*: *B. abortus*, and *B. melitensis*, and it has the same health impact as those in bovines and small ruminants, such as: abortion, stillborn calves, fetal death, reduction in milk yield and fertility [5,6]. However, according to Gwida et al. [7], brucellosis is not disease specific, and the same signs can be observed by many other infectious and non-infectious diseases. Prevalence of camelid brucellosis in several countries has been studied by Wernery [6]. He showed that the seroprevalence of this disease varies between 0.4% as a lowest prevalence reported in Chad [8] to 37.5% as a highest prevalence reported in some areas in the Sudan [9].

In order to diagnose the camelid brucellosis, bacterial culture and isolation is the gold standard, however, this method has a limited sensitivity, is laborious, time-consuming, and it is impractical to be used for a high number of samples [10]. Therefore, serological based test methods such as Rose Bengal test (RBT), Complement Fixation test (CFT), Serum Agglutination test (SAT), Indirect and Competitive Enzyme-Linked Immunosorbent assays (i-ELISA, c-ELISA), and Fluorescence Polarization assay (FPA), are considered the most practical methods to screen and confirm the diagnosis of this disease. Several studies showed differences in the sensitivity and specificity of these tests when applied on the same sera. Gwida et al. [7] (2011), showed that for a total of 895 camel serum samples, FPA had the highest number of positive results (79.3%) followed by CFT (71.4%), RBT (70.7%), SAT (70.6%) and c-ELISA (68.8%). Contrary to these findings, Khan et al. [11] claimed that the FPA performed the worst among CFT and Camel Brucella Rapid Ab test.

In this study, different known serological tests, namely RBT, c-ELISA, and FPA, and a new rapid Immunochromatographic test (ICT) were compared to assess the sensitivity and specificity of these tests in detecting *Brucella* infection in dromedary camels (*Camelus dromedarius*).

## 2. Materials and Methods

### 2.1. Ethics Statement

Samples were collected by staff of the animal Health Division of the Abu Dhabi Agriculture and Food Safety Authority (ADAFSA) for brucellosis diagnosis. Consent for sample collection was verbally obtained from animal owners. The experimental design of the study was approved by the ethical committee of ADAFSA on 26 March 2019 with reference number ADAFSA-EA-02-2019.

### 2.2. Samples

A total of 478 serum samples were collected from dromedary camels (*Camelus dromedarius*) which were suspected to be brucellosis-infected during the period 2016–2017. After bleeding, samples were kept at 4 °C and dispatched to the Veterinary Laboratories of Abu Dhabi Agriculture and Food Safety Authority (ADAFSA), Abu Dhabi, United Arab Emirates. For each sample, the serum was stored at −20 °C until performing this study.

### 2.3. Serological Tests

All the serum samples were tested by:

#### 2.3.1. Rose Bengal Test (RBT)

RBT was performed using a standard Brucella abortus antigen (Synbiotics, Lyon, France) following the provisions of the Manual of Diagnostic Tests and Vaccines for Terrestrial Animals of the World Organization for Animal Health [10]. The RBT was performed in all samples, monitoring the results with standard positive and negative controls. The presence or absence of a visible agglutination within four minutes from the start of the reaction was considered as indicative for the presence or absence of antibodies in the samples tested.

#### 2.3.2. Competitive ELISA (c-ELISA)

A competitive ELISA test was performed using a reagent kit manufactured by APHA, Addlestone- Surrey, UK, and according to the manufacturer’s instructions. The optical density (OD) values were measured at 450 nm wavelength in a microplate reader (Thermo Scientific, Waltham MA, USA). Any sample having OD value of equal or lower than 60% of conjugate control wells was taken as positive sample, otherwise negative.

#### 2.3.3. Fluorescence Polarization Assay (FPA)

The FPA was performed according to the provisions of the Manual of Diagnostic Tests and Vaccines for Terrestrial Animals of the World Organization for Animal Health [10]. FPA is a qualitative test which uses a Fluorescence Polarization technology to identify the presence of antibodies in serum, plasma or milk samples. The brucella S antibody test kit B1001 (FPA) produced by Ellie LLC, Wauwatosa, WI, USA, was used to identify in sampled sera the presence of antibodies against species of genus *Brucella* which produce smooth colonies (e.g., *B. melitensis*, *B. abortus* or *B. suis*). The test uses an O-polysaccharide (OPS) extracted from *B. abortus* and labeled with fluorescein. When antibodies against smooth *Brucella* are present in a serum, a fluorescent complex will be formed between antigen and antibody, this will change the capacity of the serum to polarise the light, and the results, in terms of difference with the original blank/background read, are expressed in milli-polarization units (mP) [12].

The results of the samples were interpreted by calculating the difference in milli-polarization units (ΔmP) of each sample using the formula: ΔmP = (Sample mP − Avg Neg control mP). The sample was considered as positive if ΔmP value was higher than 1, where the cut-off value was set at 95 mP using (ROC) analysis obtaining optimum sensitivity and specificity.

#### 2.3.4. Immunochromatography Test (ICT)

The test was performed using the Anigen Rapid Ab kit (BIONOTE, Gyeonggi, Korea). The test is a chromatographic immunoassay for the qualitative detection of *brucella melitensis*, *abortus* or *suis* antibodies in camel’s serum, whole blood or milk. 20 µL of camel serum samples were mixed with 120 µL of assay diluent, then the Immuno Chromatographic Test strips were inserted into the tubes containing the mixture and left for 15 min. Each strip has a control window (C) and a test window (T). The presence of two purple color bands inside the device window (C) and (T) demonstrate a positive result. The test result was considered negative when only the device window (C) showed purple color, whereas it was considered invalid when no color was seen in both the test (T) and the control (C) windows.

### 2.4. Testing Sensitivity by Determining Limit of Detection (LOD) for the ICT in Comparison to RBT and c-ELISA

Five routine samples which were confirmed positive by RBT were selected and diluted in twofold steps in phosphate buffered saline. Undiluted and dilutions from 1 log_2_ to 10 log_2_ were tested by ICT, RBT and c-ELISA.

### 2.5. ICT Species-Specificity

The ICT Rapid Camel Brucella Ab test kit was designed to work only with camel sera. To test this species-specificity, five RBT positive sera from camels, sheep, goats and cattle were tested by the ICT as described above.

### 2.6. Statistical Analysis

To assess the values of sensitivity and specificity of the four testing methods, a Bayesian model based on Markov Chain Monte Carlo (MCMC) has been developed. The JAGS (Just Another Gibbs Sampler) package [13] has been used and run through R Project [14] or 10,000 iterations (chains = 4; adaptation and burn-in iterations to reach the variables converged = 4000). A model for independent samples was used.

## 3. Results

### 3.1. Comparative Performance of Four Serological Tests for the Diagnosis of Dromedary Brucellosis

Table 1 shows the results of testing 478 camel sera by four serological methods.

### 3.2. Sensitivity and Specificity

The model estimated the following sensitivity (Se) and specificity (Sp) median values (and 95% lower-upper credibility levels). ICT: Se = 98.67% (95% C.L: 94.36–99.99%), Sp = 99.06% (95% C.L: 98.34–99.64%); c-ELISA: Se = 94.94% (95% C.L: 88.25–99.45%), Sp = 99.79% (95% C.L: 99.40–99.99%); RBT: Se = 68.95% (95% C.L: 56.55–80.69%), Sp = 99.92% (95% C.L: 99.64–100%); and FPA: Se = 95.05% (95% C.L: 88.23–99.51%), Sp = 99.91% (95% C.L: 99.61–100%).

In Table 2 summary statistics on results of the MCMC model are reported.

**Figure 1:**
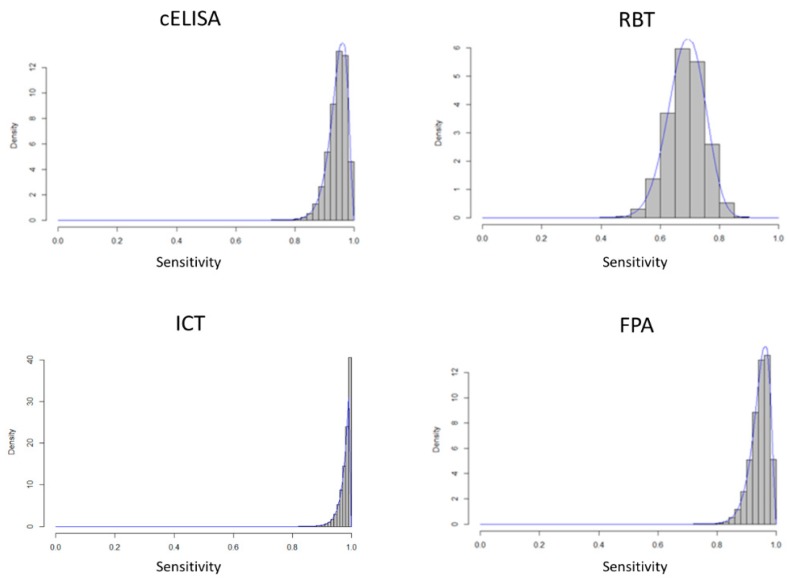
Estimated distributions of sensitivity values for c-ELISA, Rose Bengal Test (RBT), Immunochromatographic Test (ICT)and Fluorescence Poarization Assay (FPA).

**Figure 2:**
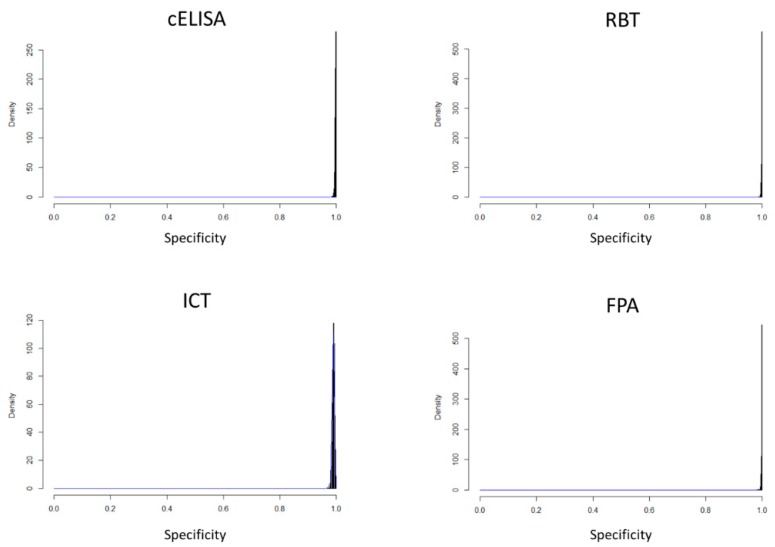
Estimated distributions of specificity values for c-ELISA, Rose Bengal Test (RBT), Immunochromatographic Test (ICT) and Fluorescence Polarization Assay (FPA).

In Figure 1 and Figure 2 the density distributions of the estimated values of sensitivity and specificity for the four types of tests are reported.

### 3.3. Limit of Detection (LOD)

**Figure 3:**
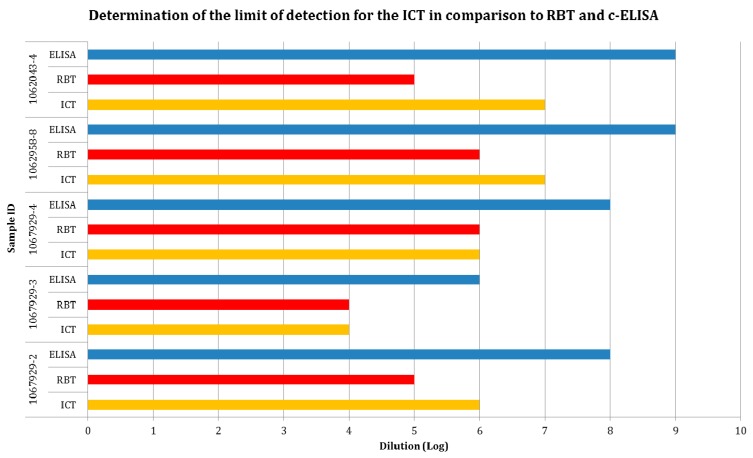
Determination of the limit of detection for the Immunochromatographic Test (ICT) in comparison to Rose Bengal Test (RBT) and c-ELISA.

Test sensitivity based on determining limit of detection (LOD) for the ICT in comparison to RBT and c-ELISA is shown in Figure 3. The mean values of the antibody titer of the five sera when tested by ICT, RBT and ELISA were 5, 4.2 and 7, respectively.

## 4. Discussion

It is well known that the possibility of designing and implementing any control program for livestock infectious diseases, including zoonoses, depends on the availability of effective diagnostic methods able to isolate and identify the causative agent or to detect the immunological response to the infection. Laboratory diagnostic tests are selected for different goals: as confirmatory methods, for screening or prevalence studies, as methods for the official certification for animal trade, and, in countries where the pathogen of concern has been eradicated, also for the demonstration of the free status or in the framework of early warning systems. The choice of the most appropriate diagnostic method for each specific objective must be based on a good knowledge of the performances of these tests under various and in-field conditions [15].

The performance of several serological tests for brucellosis are quite known in bovine animals and small ruminants [16], but it continues to be an issue, particularly in species such as camels.

Though not known to be the primary hosts, camels are highly susceptible to *B. melitensis* and *B. abortus*. Serological tests may represent an essential tool for the diagnosis and surveillance of camel brucellosis; however, concerns arise in the scientific community regarding the direct transposition from cattle and small ruminants without adequate validation.

The present study was made to compare the performances of four serological tests for the diagnosis of brucellosis in dromedary camels (*Camelus dromedarius*). This research was primarily aimed at evaluating the ICT for the serological diagnosis of brucellosis and to find the most reliable serological methods for routine camel brucellosis diagnosis in veterinary labs.

RBT has been used for serological testing in all animal species, and it is a serological method recommended by the World Organization for Animal Health (OIE) as prescribed tests for international animal trade. Its simplicity makes it an ideal screening test for small laboratories with limited resources. However, this test has several limitations including false unspecific agglutination reactions, low sensitivity particularly in chronic cases, and relatively low specificity [11,17].

Immunochromatographic tests are rapid visually read tests widely used for the diagnosis of physiological conditions, infectious and non-infectious diseases.

In comparison to the RBT, the ICT is considered to have no cross reactions, rapidly accomplished within 20 min, applicable to different samples including serum, whole blood and raw milk and it can be performed in the field. These advantages make it a possible tool on which a policy of test and slaughter could be based and put in place. We analyzed the results using a Bayesian Model which estimated the sensitivity and specificity of the ICT as 98.67% and 99.06% respectively, compared to 95.05% and 99.91% for the FPA, 94.94% and 99.79% for the c-ELISA and 68.95% and 99.92% for the RBT. In terms of sensitivity, our results suggest that the ICT shows the higher value of sensitivity, followed by the FPA, then the c-ELISA and, finally, the RBT. It is quite difficult to explain the low sensitivity values observed for RBT in this study. It is well known that higher values can be expected for bovine animals and small ruminants [15]. Further investigations should be performed on blood samples taken from camels under various epidemiological conditions. On the other hand, our study revealed no significant difference in terms of specificity between the abovementioned tests, which ranged between 99.06% for the ICT to 99.92% for the RBT. Accordingly, the ICT was found having comparable performances to the majority of tests commonly used for camel brucellosis. In a comparative study on serodiagnosis of bovine brucellosis, Eleragi et al. [18] showed that the ICT (also from BIONOTE) gave a relatively higher specificity (80%), but lower sensitivity (59.26%) compared to RBT.

A major advantage is that the Anigen Camel Brucella Rapid Ab Test Kit can be used to detect Brucella antibodies in serum, plasma, whole blood or milk, expanding the range of samples that can be used to diagnose brucellosis in camels.

In another study in dromedary camels, the Anigen Camel Brucella Rapid Ab test showed a sensitivity of 97.96% compared to CFT (98.98%), and FPA (84.69%) [11]. Furthermore, determination of limit of detection (LOD) performed in the present study have shown that the ICT is comparable to the RBT, but less sensitive than the c-ELISA. Therefore, our data, in accordance with those obtained in the previous study conducted by Khan et al. [11], confirmed good performance of the ICT methodology in the sero-diagnosis of camel brucellosis. According to Khan et al. [11], the FPA showed the lower sensitivity among the three investigated (84.69%).

## 5. Conclusions

The ICT camel brucellosis test in camel sera evaluated in the present study was confirmed to be rapid and easy to perform without requiring special equipment. Our data confirmed higher sensitivity and specificity of the ICT which should be a useful aid in the diagnosis of camel brucellosis. Finally, the ICT was found to be comparable in terms of sensitivity and specificity with the most commonly used tests for camel brucellosis.

## Figures and Tables

**Table 1:** Result of testing 478 dromedary camel sera for *Brucella* antibodies using four serological methods.

Total No. of Samples	Result	c-ELISA	Rose Bengal Test	Immunochromatographic Test	Fluorescence Polarization Assay
478	Positive	51	36	59	50
	Negative	427	442	419	428

**Table 2:** Results of the MCMC model—Summary statistics.

Category	Lower 95% C.L	Median (%)	Upper 95% C.L	Mean (%)	Autocorrelation Index (Calculated on 10 Subsequent Nodes of Each Chain)
Sensitivity c-ELISA	88.25	94.94	99.45	94.39	0.0022
Specificity c-ELISA	99.40	99.79	99.99	99.75	−0.0038768
Sensitivity RBT	56.55	68.94	80.69	68.72	−0.0051273
Specificity RBT	99.64	99.91	100	99.88	0.0049039
Sensitivity ICT	94.36	98.67	99.99	98.09	−0.0055805
Specificity ICT	98.34	99.06	99.64	99.02	0.0024034
Sensitivity FPA	88.23	95.05	99.51	94.49	−0.006178
Specificity FPA	99.61	99.91	100	99.87	0.0004585
